# Improving small RNA-seq by using a synthetic spike-in set for size-range quality control together with a set for data normalization

**DOI:** 10.1093/nar/gkv303

**Published:** 2015-04-13

**Authors:** Mauro D. Locati, Inez Terpstra, Wim C. de Leeuw, Mateusz Kuzak, Han Rauwerda, Wim A. Ensink, Selina van Leeuwen, Ulrike Nehrdich, Herman P. Spaink, Martijs J. Jonker, Timo M. Breit, Rob J. Dekker

**Affiliations:** 1 RNA Biology & Applied Bioinformatics research group, Swammerdam Institute for Life Sciences, Faculty of Science, University of Amsterdam, Amsterdam 1090 GE, The Netherlands; 2 Netherlands eScience Center, Amsterdam 1098 XG, The Netherlands; 3 Department of Molecular Cell Biology, Institute of Biology, Leiden University, Gorlaeus Laboratories - Cell Observatorium, Leiden 2333 CE, The Netherlands

## Abstract

There is an increasing interest in complementing RNA-seq experiments with small-RNA (sRNA) expression data to obtain a comprehensive view of a transcriptome. Currently, two main experimental challenges concerning sRNA-seq exist: how to check the size distribution of isolated sRNAs, given the sensitive size-selection steps in the protocol; and how to normalize data between samples, given the low complexity of sRNA types. We here present two separate sets of synthetic RNA spike-ins for monitoring size-selection and for performing data normalization in sRNA-seq. The size-range quality control (SRQC) spike-in set, consisting of 11 oligoribonucleotides (10–70 nucleotides), was tested by intentionally altering the size-selection protocol and verified via several comparative experiments. We demonstrate that the SRQC set is useful to reproducibly track down biases in the size-selection in sRNA-seq. The external reference for data-normalization (ERDN) spike-in set, consisting of 19 oligoribonucleotides, was developed for sample-to-sample normalization in differential-expression analysis of sRNA-seq data. Testing and applying the ERDN set showed that it can reproducibly detect differential expression over a dynamic range of 2^18^. Hence, biological variation in sRNA composition and content between samples is preserved while technical variation is effectively minimized. Together, both spike-in sets can significantly improve the technical reproducibility of sRNA-seq.

## INTRODUCTION

Small RNA (sRNA) is commonly defined as the fraction of the transcriptome that contains all RNA molecules shorter than 200 nucleotides (1). This class of cellular molecules includes for example: micro-RNA, miRNA (2); piwi-interacting RNA, piRNA (3); transfer RNA, tRNA (4), 5S and 5.8S ribosomal RNA, rRNA (5); small nuclear RNA, snRNA (6) and small nucleolar RNA, snoRNA (7). Being highly diverse in length, structure and expression, sRNAs have been described to perform a wide variety of functions ranging from post-transcriptional regulation (8) and silencing of mobile genetic elements (9), to processing of pre-mRNA (10) and post-transcriptional modifications (11). However, the exact function and biological relevance of most known sRNA species is largely unknown. Yet, since many sRNAs are emerging as essential regulators of numerous biological processes, there is an increasing desire to study their expression behavior in parallel with that of mRNAs and long-non-coding RNA. As microarray and qPCR are less suitable methods to study sRNAs, next-generation sequencing (NGS) has quickly become the standard technology to measure sRNA expression levels (sRNA-seq) (12). When using sRNA-seq for sRNA expression analysis, there are currently two main challenges that need to be dealt with: (i) how to check the size range and distribution of isolated sRNAs between samples, given several sensitive size-selection steps in the protocol and (ii) how to optimally normalize data between samples given the low complexity of sRNA types. Because both issues are essential for accurate quantification of sRNA expression differences between biological samples, they have to be addressed properly.

Standard sRNA-seq protocols contain some form of size selection at one or more steps in the procedure, even when starting from total RNA. The common protocols that are being used to isolate sRNA for input in sRNA-seq enrich for RNA molecules in a limited range of roughly 15–100 nucleotides. These protocols are relatively unstable, often due to (undetected) contaminants in the samples or subtle changes in the final ethanol concentrations, which may result in sRNA isolation over a different size range or with a different efficiency per given size between samples. Such variations are undesirable and could erroneously be interpreted as the result of differential sRNA expression. As such, the performance of the size-selection steps has to be checked with proper controls. The best approach would be to design synthetic spike-in controls that cover the protocol's relevant sRNA size range and add these to all samples at the beginning of the procedure.

Expression-data normalization aims at minimizing variation that is not the result of biological effects and is thus essential for detecting the biologically relevant differences in gene expression (13). This is similar for both RNA-seq and sRNA-seq, with an important difference being that sRNA-seq involves low complexity samples in which sometimes as few as ten transcripts make up 50% of all reads in a sRNA sample (this study). Hence, statistical normalization approaches that are frequently used in RNA-seq data analysis, such as global normalization using the total number of (mapped) reads (14,15), should be applied to sRNA-seq with the utmost caution (13,16,17). In particular when they rely on the assumption that the number of differentially expressed sRNAs is negligible compared to the size and complexity of the small transcriptome. Especially for sRNA, scaling on the number of obtained sequencing reads can be problematic if cellular RNA content and composition are significantly different between tissue types or have changed drastically as a result of the biological stimulus or condition under study (18). Under such circumstances of global gene-expression shift, the relative quantitative comparison of transcriptomes will be subject to substantial bias. Another RNA-seq normalization strategy is to use an internal biological reference that is constant over all samples within a given study. So far, universal internal sRNA references have not been discovered and are unlikely to exist. Given the low complexity and high variability in sRNA samples, the best normalization approach would therefore be based on an external reference that is established by adding synthetic spike-ins at a constant spike-in to total RNA ratio. This is different from the internationally accepted standard spike-in control set (ERCC) that was primarily designed to inspect data-normalization procedures (19) and is, as of recently, cautiously being used to normalize RNA-seq data (20). In any case, the ERCC set only contains RNA controls that are too large (>250 nucleotides) for sRNA-seq (19), so a new set of small spike-ins is needed for the normalization of sRNA-seq data.

Therefore, to address these sRNA-seq issues, we developed, implemented and tested two dedicated external spike-in sets for use in sRNA-seq experimentation: one set of size spike-in controls to monitor size bias in the entire sRNA-seq procedure (sRNA-seq size-range quality control; SRQC) and another set of data-normalization spike-ins for use as an external reference in the normalization of sRNA-seq expression data (sRNA-seq external reference for data normalization; ERDN). In summary: with these two spike-in sets, we are able to evaluate the quality and size-range of the RNA size-selection and perform effective data normalization in sRNA-seq.

## MATERIALS AND METHODS

### Biological materials

Adult zebrafish (strain ABxTL) were handled in compliance with local animal welfare regulations and maintained according to standard protocols (http://zfin.org). The breeding of adult fish was approved by the local animal welfare committee (DEC) of the University of Leiden, The Netherlands. All protocols adhered to the international guidelines specified by the EU Animal Protection Directive 86/609/EEC. Adult zebrafish and oocytes were flash-frozen in liquid nitrogen and stored at −80°C. Before freezing, fish were put under anesthesia using 0.02% buffered 3-aminobenzoic acid ethyl ester (Tricaine).

### Total RNA and sRNA isolation

Whole zebrafish or isolated tissues were pulverized in liquid nitrogen with a mortar and pestle, after which sRNA isolation was performed using the miRNeasy Mini Kit (Qiagen). In brief, powdered tissue was homogenized in TRIzol Reagent (Life Technologies) and 1-bromo-3-chloropropane (BCP) was added. After centrifugation RNA partitioned to the upper aqueous phase, which was carefully removed and subjected to column-based sRNA isolation according to the manufacturers’ instructions. RNA concentration was measured on a NanoDrop ND-2000 (Thermo Scientific) and RNA integrity was examined on a 2200 TapeStation instrument using R6K and High Sensitivity R6K ScreenTapes (Agilent Technologies).

### Design of the data-normalization and size-range quality control spike-in sets

A list of candidate random RNA sequences of the desired length was generated using the following constraints: maximum homopolymer length of two nucleotides, GC-content between 40 and 60%, Δ*G* for hairpin formation >−0.5 kcal/mol at 37°C (UNAFold: http://mfold.rna.albany.edu/), and an *E*-value >10 when aligned against the NCBI nucleotide collection (nr/nt: http://blast.ncbi.nlm.nih.gov) using BLASTN. From these candidates 19 sequences of 25 nucleotides long were selected for use as external reference for data-normalization (ERDN) spike-ins. For size-range quality control (SRQC) spike-ins different sequences were selected of 10, 16, 19, 22, 25, 28, 34, 40, 50, 60 and 70 nucleotides long. The 10-nucleotide SRQC spike-in was not checked using BLASTN because of its short size. These sequences were obtained as single-stranded oligoribonucleotides with a 5′-phosphate and polyacrylamide gel purified (Eurogentec S.A.). The oligoribonucleotides were dissolved in 100 μM in RNase-free TE (10 mM Tris–HCl pH 8 and 1 mM ethylenediaminetetraacetic acid (EDTA)) and stored in aliquots at −80°C.

### Next-generation sequencing

sRNA libraries were prepared according to the manufacturers’ protocols using the Ion Total RNA-Seq Kit v2 (Life Technologies). Briefly, adapters were ligated to the sRNA and a reverse transcription reaction was performed. The resulting cDNA was amplified and at the same time barcoded with IonXpress RNA-Seq BC01-BC16 (Life Technologies). The yield and the size distribution of the amplified cDNA were assessed using the 2200 Tapestation with the Agilent D1K ScreenTape (Agilent Technologies). Emulsion PCR was performed using the Ion PI Template OT2 200 Kit on an Ion OneTouch 2 Instrument, after which the template-positive Ion PI Ion Sphere Particles were recovered, quantified with a Qubit 2.0 ﬂuorometer, and enriched using an Ion OneTouch ES (Life Technologies). Sequencing was carried out on the Ion Proton system using an Ion PI Chip v2 and Ion PI Sequencing 200 kit (Life Technologies) following the manufacturers’ protocols (Revision 3.0).

### Data access

All sequencing results are accessible through the European Nucleotide Archive (http://www.ebi.ac.uk/ena/) under the project accession number ERP007147. For detailed info see Supplementary Data 4.

### Bioinformatics analysis

#### Mapping NGS reads to spike-in sets

Before alignment of the reads to the spike-in sets, all reads were trimmed to 40 nucleotides from the 3′-end using Trimmomatic 0.30 (21) (option CROP:40). Bowtie2 (22) was used for the alignment of the trimmed reads to the synthetic spike-in sequences. The parameters used for alignment were -L 6 -i S,0,0.5 –ignore-quals –norc –score-min L,-1,-0.6 -D 20. This corresponds to ∼10% of mismatches allowed. Samtools (23) was used to convert the alignment results to the BAM file format. Reads were selected only if the alignment length was >80% of the target length using the Rsamtools package (24).

#### Mapping NGS reads to sRNA

For miRNA alignment, all reads were trimmed to 30 nucleotides and reads shorter than 15 nucleotides were discarded. Trimmed reads were aligned to the mature zebrafish miRNA sequences from miRBase version 20 (25–29) with Bowtie2 using the same settings as used for spike-in sequences alignment. Only reads with perfect alignments were selected.

For piRNA alignment, all reads were trimmed to 40 nucleotides and reads shorter than 12 nucleotides were discarded. Trimmed reads were aligned to both strands of piRNA sequences from piRNABank (30) with Bowtie2 using the same alignment score settings as used for spike-in sequences alignment. Only reads with perfect alignments were selected. Finally, Samtools (23) was used to convert the alignment results to the BAM file format.

#### Normalization

For testing the SRQC spike-in set using different ethanol concentrations (Figure 2A, Supplementary Figure S1.1 and Supplementary Figure S2.3) the number of reads that mapped to each size spike-in was first divided by the total number of zebrafish miRNA reads for that sample and then multiplied by the average number of mapped miRNA reads over all samples.

**Figure 1.**
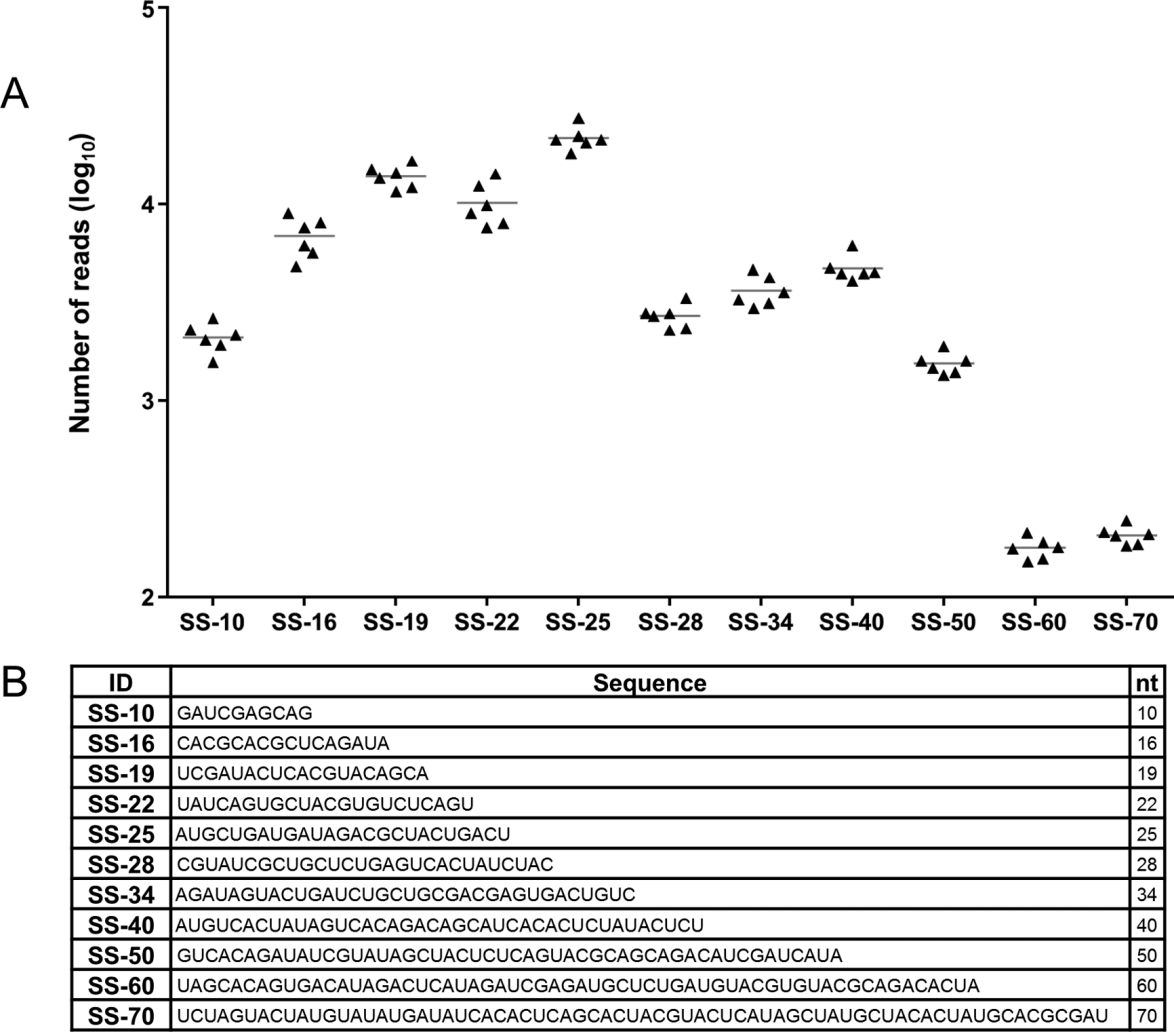
Design and testing of small-RNA size-range quality control (SRQCs) spike-in set. (**A**) *Technical reproducibility*. sRNA-seq analysis over six independent SRQC spike-in experiments. Triangles in the dot plot represent the non-normalized read count of six technical replicates for each SRQC (SS-10 to SS-70). (**B**) *SRQC sequences*. Size spike-ins (SS-) of 10 to 70 nucleotides long.

**Figure 2.**
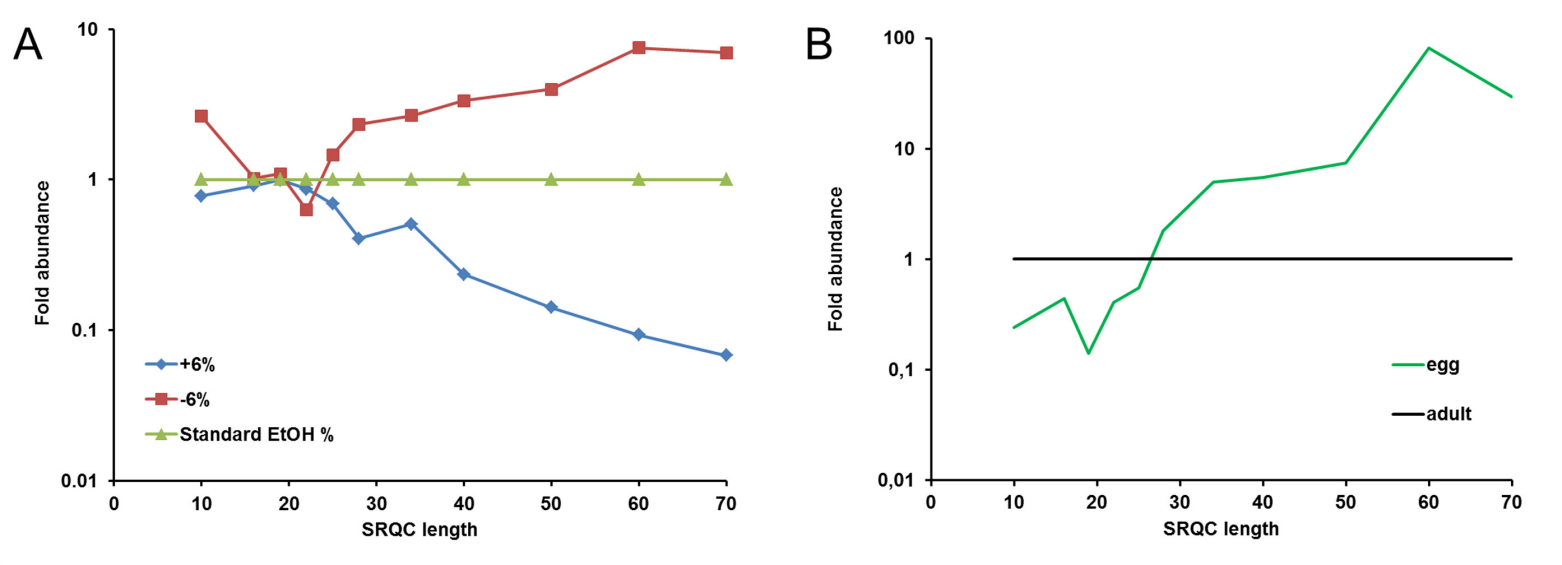
Using SRQCs to monitor size range in sRNA-seq. (**A**) *Monitoring size-range biases in sRNA isolation*. Fold abundance here is for each size spike-in the ratio of the observed number of reads divided by the number of reads found when using the standard ethanol concentration. (**B**) *An example of biased size range in sRNA-seq*. Fold abundance here is for each size spike-in the ratio of the observed number of reads divided by the number of reads in the adult male zebrafish sample.

ERDN-based normalization (Figures 2B, 4, 5C–F) was performed by first calculating size factors from the ERDN counts using the DESeq R package (31). These size factors were then used to scale the number of reads between the samples. By this approach all ERDN spike-ins contribute in the normalization procedure, irrespective of their abundance. For comparison, this normalization procedure was repeated using the miRNA-mapped reads as input to calculate the size factors in DESeq.

**Figure 3.**
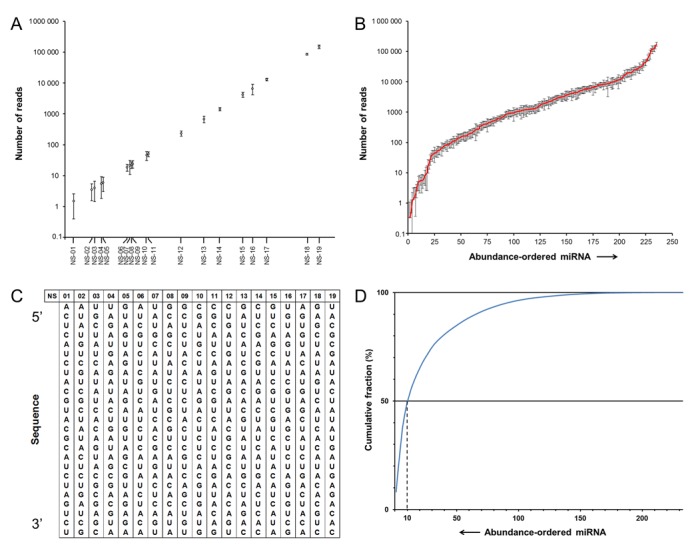
Design and testing of external small-RNA normalization spike-ins (ERDNs). (**A**) *Technical reproducibility*. sRNA-seq analysis of six independent ERDN spike-in experiments. The average non-normalized number of reads for each normalization spike-in plotted against the median number of reads of each spike-in over the six technical replicates. Error bars indicate standard deviations. (**B**) *Dynamic range of miRNA expression in zebrafish*. Zebrafish miRNAs are shown, sorted by increasing abundance and plotted against their corresponding average number of reads over the six replicates. Error bars are standard deviations (*n* = 6). (**C**) *ERDN sequences*. Oligoribonucleotide sequences of the 25-mer ERDN spike-in set. (**D**) *Quantitative diversity of the zebrafish miRNA pool*. Zebrafish miRNAs are shown sorted by decreasing abundance and plotted as cumulative fraction of reads that is consumed by each miRNA. The dashed line indicates the fraction of the reads used up by the 10 most abundant miRNAs.

**Figure 4.**
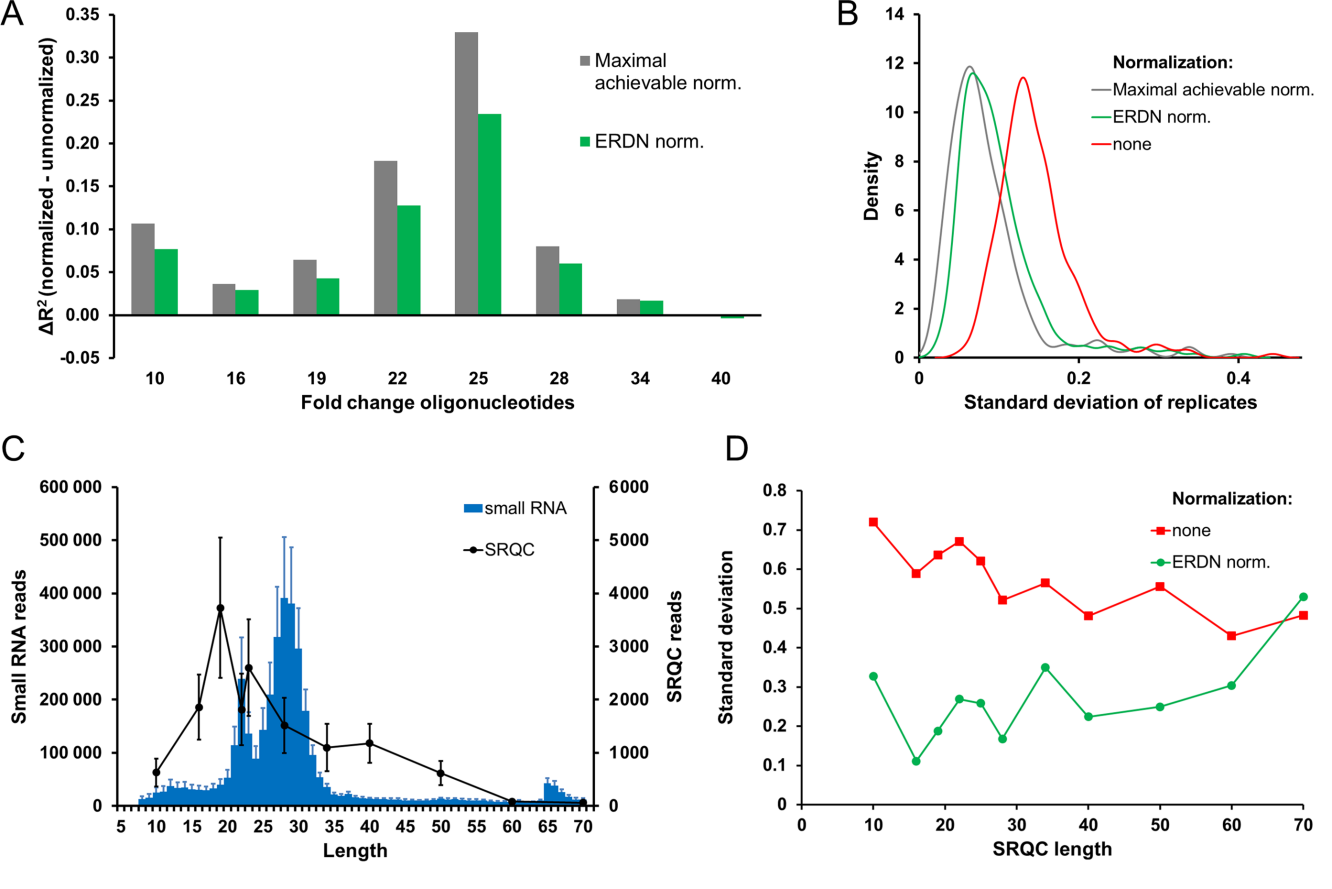
Evaluating ERDN-based normalization. (**A**) *Effect of ERDN-based normalization on the dilution data of the fold-change oligoribonucleotides*. Linear regression analysis was performed on plots of the dilution factor versus the number of reads obtained for the 2-fold serial dilutions of each fold-change oligonucleotide. The difference in the coefficients of determination (Δ*R*^2^) between non-normalized data and DESeq-normalized data is plotted for each fold-change oligo (SS-10 to SS-40). DESeq normalization was performed using as a reference either the number of miRNA-mapped reads (green) or the ERDNs (blue). SS-50, SS-60, SS-70 are not showed as they have <10 reads in more than three runs. (**B**) *Effect of ERDN-based normalization on the variance of replicate miRNA samples*. The standard deviation of the number of reads (log_2_) obtained for each zebrafish miRNA was calculated over the eight replicates used under (A). The results are presented in kernel density plots where the density (vertical axis) signifies the number of miRNAs that have a particular standard deviation (horizontal axis). Red, not normalized; green, normalized with total miRNA-mapped reads, and blue, normalized with ERDN. (**C**) *Average raw read distribution of small RNA and SRQC*. The average non normalized number of reads of the six different samples were calculated and plotted against the length of sRNAs (blue, left *y*-axis) and SRQC spike-ins (black, right *y*-axis). The error bars show the standard deviation. (**D**) *Effect of ERDN-based normalization on the variance of sequences of different length*. The standard deviation of the number of reads (log_2_) obtained for each size spike-in was calculated over six female zebrafish samples, for non-normalized data and ERDN-normalized data.

**Figure 5.**
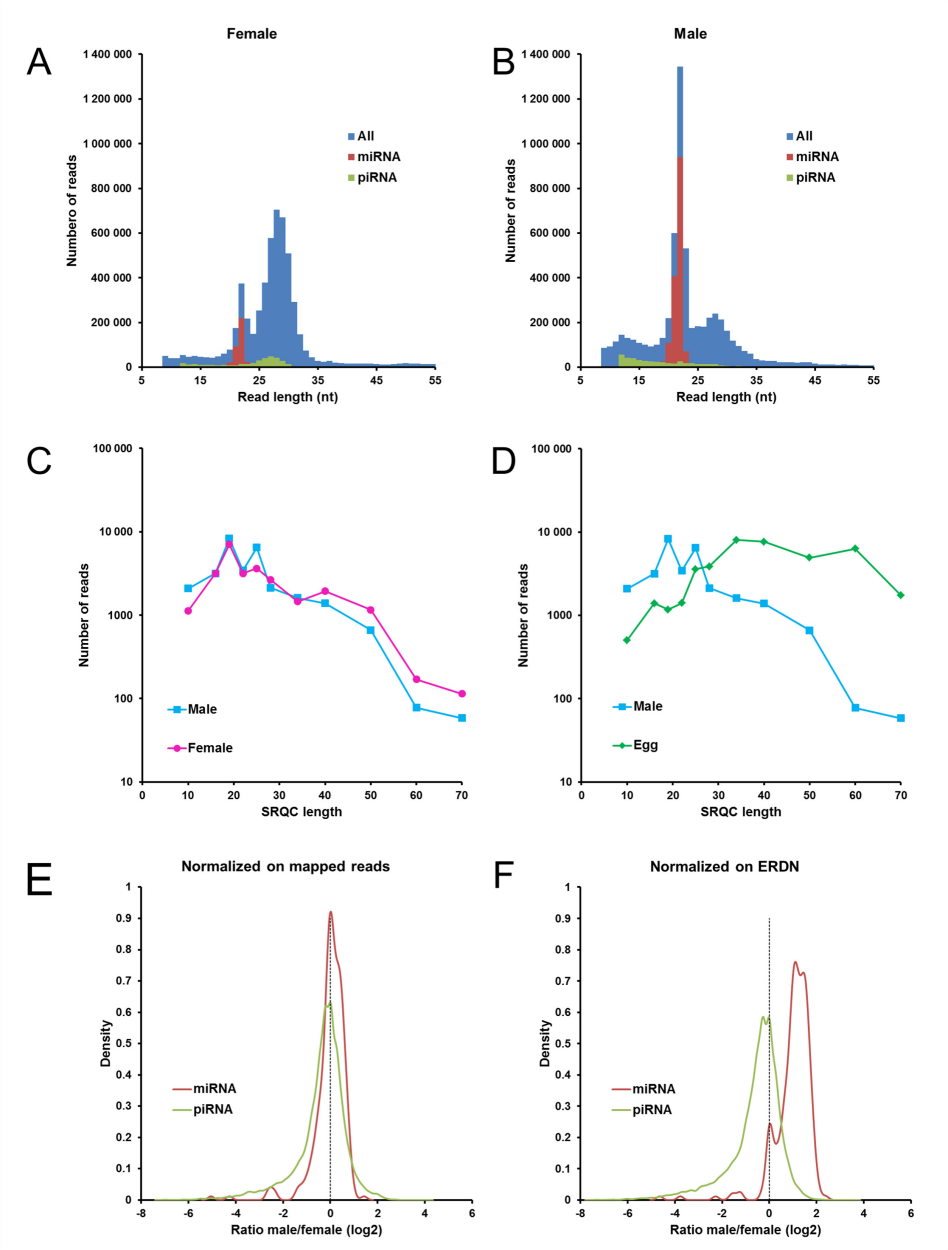
Using ERDN for normalization of samples with different miRNA content. (**A** and **B**) *Size distribution of sRNA in female and male adult zebrafish*. Histograms of the number of reads versus the read length are shown for a female (A) and a male (B) adult zebrafish. *Blue*, all reads; *red*, miRNA-mapped reads and *green*, piRNA-mapped reads. Reads shorter than nine nucleotides are not included. (**C** and **D**) *Size-selection profile in zebrafish samples*. Comparison of the SRQC reads between the female and male adult zebrafish (C) and between male and egg (D). (**E** and **F**) *ERDN-based normalization preserves the natural miRNA content*. Reads were normalized by DESeq using as a reference either the number of mapped reads (E) or the ERDN spike-in set (F). For each miRNA and piRNA, the average number of reads was calculated over four female and over four male zebrafish. The log_2_ ratio of male over female miRNAs (red) and piRNAs (green) is displayed in kernel density plots. The vertical dashed line indicates an equal number of reads in female and male zebrafish.

#### R^2^ calculation (Figure 4A)

The levels of each fold-change control in all eight dilutions were evaluated by individually plotting their relative input concentration against their normalized number of reads. These data should follow a linear relationship on a log_2_ scale with a slope that equals the fold-change difference between consecutive dilution mixes. The difference between the observed and expected fold-changes was thus assessed by linear regression with a fixed slope of 1 (log_2_ of the expected 2-fold changes), returning the coefficient of determination (*R*^2^) as a measure of similarity.

#### Down-sampling raw sequence files

Down-sampled fastq files were generated by randomly sampling 2.5, 3.0, 3.5, 4.0, 4.5 and 5.0 million of reads from the original fastq files (starting respectively from 7 611 054, 6 343 893, 6 990 446, 6 082 600, 6 227 225 and 6 054 478 total reads) using the Seqtk tool (http://github.com/lh3/seqtk).

## RESULTS AND DISCUSSION

### Development of a size-range quality control (SRQC) spike-in set for sRNA-seq

In order to make RNA-seq libraries that are focused on sRNA (<200 nt), library preparation protocols contain size-selection steps at different stages in the NGS procedure. For example, the size range of Ion Torrent technology based NGS libraries is controlled by sRNA isolation from total RNA, followed by size selection after cDNA synthesis and after library amplification. Unfortunately, the performance of these size-selection steps can be sample-dependent, as well as sensitive to variation in lab handling. This may have a significant impact on the sequencing outcome. For this reason, we decided to design a set of external sRNA size-range quality controls (SRQC) to allow checking of the NGS size-selection steps. These controls are to be added to total RNA as early in the procedure as possible and enable routine monitoring of a consistent size selection of RNA isolation, library preparation and sequencing, from the beginning to the end of the procedure. As the standard sRNA isolation methods followed by standard sRNA-seq, investigate sRNA in the range of ∼10–70 nucleotides, we decided to design an SRQC spike-in set that consists of 11 synthetically-synthesized, single-stranded 5′-phosphorylated oligoribonucleotides of increasing length (10–70 nucleotides; Figure 1B).

The differences in sequencing efficiency between the individual spike-ins can be considerable, so we started by levelling-out most of these variances. At the same time, we sought for an appropriate concentration for each SRQC oligonucleotide that allows reliable measurement, but avoids using-up too many sequencing reads. For this purpose, an equimolar mix of all SRQC oligoribonucleotides was added to identical zebrafish sRNA samples at 9.6 × 10^8^, 9.6 × 10^9^ or 9.6 × 10^10^ total number of spike-in molecules per μg of total RNA. Next, sRNA-seq was performed, omitting all size-selection steps, resulting in 1.5, 1.1 and 1.4 million reads, respectively, with the number of reads mapping to each spike-in varying between 0.01 and 14.8% (Supplementary Figure S1.1A). The concentration of each individual spike-in was adjusted to obtain similar percentages of reads for all spike-ins in the mix (Supplementary Table S1.2). In addition, the overall concentration of the SRQC mix was chosen to be 1.9 × 10^10^ oligoribonucleotides/μg of total RNA, which will produce sufficient reads for accurate counting, while consuming at most 1–2% of the total number of reads in each sample.

### Testing the SRQC spike-in set for sRNA-seq

It is obviously extremely important for any control to be robust. Hence, to evaluate the reproducibility of the SRQC spike-in set, we performed a test experiment. Zebrafish total RNA (including sRNA) was split in six equal aliquots to which equal an amount of SRQC mix was added. On these spiked samples, a standard sRNA-seq analysis was performed, including all size-selection steps. The results show that the technical reproducibility of the spiking and sequencing procedure is high (Figure 1A). Although the SRQC spike-in set reveals a standard sRNA-seq size range from ∼10 to 50 nucleotides, it is evident that the sRNA-seq procedure is optimized for sequences between 16 and 25 nucleotides long, i.e. the miRNA range (Figure 1A). Based on these findings, we concluded that the reproducibility of SRQC spiking and sequencing is sufficient for monitoring the quality of the size selection in the sRNA-seq procedure.

### Applying the SRQC spike-in set in sRNA-seq

Next to reproducibility, sensitivity is another important issue of experiment controls. To investigate this for the SRQC spike-in set, we designed an experiment in which we manipulated the size-selectivity range of the sRNA isolation protocol. In the Ion Torrent sRNA-seq workflow (Supplementary Figure S2.1), it is recommended to first isolate sRNA by removing large nucleic acids (>200 nucleotides) from total RNA using a low ethanol concentration in combination with silica-based spin columns. Changing the ethanol concentration affects the sRNA isolation size range. Therefore, zebrafish total RNA was spiked with the SRQC mix, split into equal aliquots and subjected to sRNA isolation using either: standard, standard plus 6% or standard minus 6% ethanol concentrations. sRNA-seq analysis of the isolated sRNA samples showed a clear effect of ethanol on the SRQC read counts (Figure 2A). At a higher ethanol concentration, an increased number of reads was observed for the longer spike-ins, while at a lower ethanol concentration the opposite effect was observed. In a similar fashion, we have altered all other size-selection steps in the library preparation protocol (Supplementary Table S2.2) using alternative ethanol concentrations. The size range profiles that were obtained again demonstrated the ability of the SRQCs to detect size-range selection biases anywhere in the sRNA-seq procedure (Supplementary Figure S2.3).

To test our SRQC spike-in set in a real-life scenario, we evaluated the isolation of sRNA from two different biological samples. It is our experience that the standard sRNA isolation protocol performs differently when using zebrafish oocytes as compared to somatic zebrafish tissues, presumably due to impurities from the oocytes’ chorion. Hence, total RNA from adult zebrafish (whole body) and oocytes was spiked at a fixed SRQC/total RNA ratio and analysed by sRNA-seq. The size range that was shown by the SRQCs revealed a marked loss of spike-ins smaller than 28 nucleotides in the sRNA fraction from oocytes compared to the adult (Figure 2B). This means that scientists who want to analyse for instance the miRNA population of oocytes using a standard sRNA isolation protocol might erroneously conclude that there are only few miRNAs in an oocyte.

In conclusion, these findings demonstrate that the SRQCs can be used to reliably detect differences in the size selection of sRNA-seq. We recommend including SRQCs in each sample to enable routine quality control monitoring of the sRNA-seq procedure for undesirable size-range biases that might occur anywhere in the RNA isolation and/or sequencing procedures. Size-range variations between samples will result in noisy expression data, thereby frustrating the reliable identification of e.g. differentially expressed miRNAs. Lastly, we believe that SRQCs can be useful for all NGS platforms that rely on size-selection steps, such as the gel-base size selection in Illumina's TruSeq sRNA-seq protocols.

### Development of an external reference for data-normalization (ERDN) spike-in set for sRNA-seq

Data normalization is an important element in omics experimentation. Between samples there are differences in technical efficiency that result in variations in the generated data. Correct normalization of gene-expression data is therefore paramount for the accurate comparison of expression levels. Normalization is typically performed with the premise that the expression of the vast majority of genes does not change. This assumption does not necessarily hold true for the different types of sRNA because of the lower complexity of the sRNA pool in comparison with a full transcriptome. As an example, we investigated the quantitative diversity of the miRNA pool in adult zebrafish (Figure 3D). The 10 miRNAs with the highest number of reads consumed ∼50% of all miRNA reads. For this reason, variations in the expression of these highly abundant miRNAs between different biological samples can have a substantial impact on the total miRNA pool, thus ruling out normalization strategies that are merely based on the (total number of) miRNA reads. In this scenario, normalization would ideally be performed using transcripts that are invariant in expression across all samples and that span the entire range of expression levels. For this purpose, we designed a dedicated set of small-RNA external-reference data-normalization spike-ins (ERDNs) to serve as such invariant references between samples. When combined with total RNA at a fixed spike-in to RNA ratio, these oligoribonucleotides can be used to normalize sRNA expression profiles based on the total RNA starting input prior to size selection.

The use of synthetic spike-ins for normalization of sRNA-seq data was previously published (32). As acknowledged by the authors, their approach was limited by the use of only three different oligoribonucleotides. Moreover, the concept was not extensively tested, nor developed further as they did not use these spike-ins in the sRNA-seq libraries in later studies (33). Another approach is using commercial miRNA mixes with spike-ins in equimolar quantity (34,35). However, these mixes are less suitable as they do not span the entire expression range, a desired characteristic for a good baseline population to be used in normalization (36). Also, as these spike-ins are identical to known miRNAs published in miRBase, they cannot be used as internal spike-ins for sRNA-seq. We therefore designed the ERDN spike-in set to include 19 single-stranded 5′-phosphorylated oligoribonucleotides of 25 nucleotides long (Figure 3C) that cover the entire expression range of sRNA. As before, we first compiled an ERDN mix in which the individual spike-ins are balanced for differences in sRNA-seq efficiency (Supplementary Figure S1.1B and Supplementary Table S1.3) and consume ∼3% of the total number of reads,

### Testing the ERDN spike-in set for sRNA-seq

Given that the normalization spike-ins will function as a fixed reference in a variable biological sample, high technical reproducibility of spike-in sequencing is essential. To evaluate this, the ERDN mix was added six times independently to equal aliquots taken from a single batch of zebrafish total RNA. The samples were individually subjected to sRNA isolation and sRNA-seq. Reproducibility was high for spike-ins with >100 reads and gradually decreased for lower read counts (Figure 3A), as was previously described for RNA-seq (37). Comparison of the number of reads that mapped to known zebrafish miRNAs (Figure 3B) demonstrated that the read distribution of the ERDN spike in set (Figure 3A) covers the entire dynamic range of miRNAs in these biological samples, i.e. from a single read to 2 × 10^5^ reads per 4 million total reads on average per technical replicate. We conclude that, from a technical perspective, the ERDN mix could be suitable for use as a reliable reference for data normalization in sRNA-seq.

In order to test this, we designed an experiment in which predetermined fold-changes were artificially introduced in zebrafish total RNA using external test spike-ins of various sizes (10–70 nucleotides; Supplementary Data 3). The effect of ERDN-based normalization on these fold-changes was assessed. After sRNA isolation and sRNA-seq the number of reads mapping to the fold-change spike-ins varied between 0 and 10^4^, thus covering a large part of the dynamic range of the ERDNs (10^5^ reads). To then evaluate the efficacy of the ERDN spike-in set in normalization, the following approach was taken: since the fold-change mixes were added to an identical background of zebrafish RNA, a scaling based on the number of miRNA reads would be the best available normalization method to compare ERDN-based normalization to. We therefore performed a data normalization using the ERDN spike-in set and evaluated how close the results would match with the absolute data normalisation based on the identical miRNA background in these samples. Subsequently, the coefficient of determination (*R*^2^) between the observed and expected fold-changes was calculated and used to check the performance of the normalization procedure. The average *R*^2^ values for all fold-change test spike-ins were 0.87 ± 0.12, 0.92 ± 0.06 and 0.91 ± 0.07 for non-normalized reads, total-number of mapped miRNA reads normalized data and ERDN-normalized data, respectively (Figure 4A). We conclude that the accuracy of the observed fold-changes significantly improves, close to the best possible normalization reference, when the ERDN spike in set is used as an external reference for data normalization.

We next took an alternative approach to analysing these data by investigating the effect of ERDN-based normalization on the variance of the replicate zebrafish miRNA measurements (*n* = 8), which represent identical technical replicates. For this purpose, the standard deviation distribution of the mapped zebrafish miRNAs was calculated for each miRNA over all eight replicates and plotted in a kernel density plot (Figure 4B). ERDN-based normalization significantly diminishes variability between replicates, almost as well as the normalization based on total miRNA-mapped reads from identical samples.

Lastly, we checked whether our ERDN-based normalization strategy would also work on RNA with sizes outside the miRNAs or piRNA size range. To that end, we used the SRQC spike-ins as representative sRNAs covering sizes from 10 to 70 nucleotides. The reproducibility of the number of SRQC reads was used as a read-out for the effectiveness of ERDN-based normalization. First, sRNA-seq data from six different female zebrafish spiked with the SRQC and ERDN mixes was randomly down-sampled to mimic an experiment with a larger variance in sequencing depth. Otherwise, the total number of reads between these samples would have been too similar (CV = 0.087) to monitor a clear improvement of SRQC reproducibility. Next, normalization based on the ERDN spike-ins was performed, showing a robust reduction of the variance of the SRQC reads over most of the tested size range (Figure 4D). The variance did not improve for 70-nucleotide spike-in after normalization, but this is likely a consequence of the low number of reads that mapped to this spike-in sequence (<100) brought about by the size-selectivity of the standard Ion Torrent sRNA-seq protocol (Figure 4C). We conclude that the ERDN spike-ins can be used to effectively normalize for variable sequencing depth over the range from 10 to 70 nucleotides.

In summary, the ERDN spike-in set yields reproducible results in the size range from 10 to 70 nucleotides and covers a dynamic range of 2^18^ while having a ∼2-fold difference in number of reads between consecutive spike-ins. ERDN-based normalization improves the fold-change response of artificial fold-change controls and improves technical reproducibility of sRNA sequencing. We suggest the use of ERDNs for sample-to-sample normalization of sRNA-seq data, in order to achieve a more accurate quantification of differential expression between biological samples.

### Applying the ERDN spike-in set in sRNA-seq

In contrast to our test experiment with identical sRNA zebrafish samples, normalization on the number of mapped reads will fail to correctly preserve information on differential expression in cases where sRNA content and composition differ significantly between biological samples. To demonstrate the applicability of ERDN-based normalization in such a scenario, we applied our normalization spike-in set in a sRNA-seq experiment comparing sRNA expression profiles of male and female zebrafish. For the analysis, we have chosen to focus on miRNA and piRNA, as these are abundant and their sequences are readily available. Total RNA was isolated from the whole bodies of four adult male and four adult female zebrafish and spiked with the SRQC and ERDN spike-in mixes at a fixed total-RNA/spike-in ratio. sRNA-seq analysis showed that the size distribution of the NGS reads is remarkably different between female (Figure 5A) and male (Figure 5B) zebrafish, with 10% versus 29% of the reads mapping to miRNAs, respectively. We turned to the SRQC spike-ins to see if this difference was caused by unequal size-selection ranges between female and male zebrafish. We previously observed a substantial difference in the size-selection performance between somatic and oocyte zebrafish samples (Figures 2B and 5D) that hampers proper analysis of sRNA-seq data. For the adult female and male zebrafish, however, the SRQC spike-in set revealed an almost identical size-range distribution, suggesting that no artificial size-based skewing between samples was introduced during the library preparation (Figure 5C). We can therefore conclude that the female samples contained a substantially smaller fraction of miRNAs compared to the male samples, probably due to the vast amount of maternal RNA originating from the oocytes. In this situation, normalization on the total number of miRNA-mapped reads would skew the results toward a levelling of the ratios between male and female samples. Indeed, density plots of the male over female miRNA ratios show that a mapped-reads based normalization centres the distribution of log_2_ ratios close to zero for both miRNAs and piRNAs (Figure 5E). In contrast, normalization using the ERDN spike in set as a reference retains the biological difference, thereby demonstrating a higher miRNA content in male zebrafish and a similar overall expression of piRNAs compared to female zebrafish (Figure 5F). Hoen *et al*. suggest to treat the small RNA population not as a whole, but as a set composed by smaller subsets such as miRNA, piRNA, etcetera and then to normalize on the number of reads of each subset (38). However, this approach was not successful in our case as shown before. That might be due to the fact that Hoen *et al*. checked for variability through identical replicates sequenced in different laboratories, while in our case we compared two biologically very different samples. Together, these findings prove that normalization on subsets mapped reads is not suitable in experiments, where the composition of the sRNA pool is different between samples. The addition of ERDN spike-ins to total RNA samples at a fixed ratio, however, effectively enables normalization relative to the amount of starting total RNA. Hence, biological variation in sRNA composition and content between samples is preserved while at the same time technical variation is effectively minimized.

## SUPPLEMENTARY DATA

Supplementary Data are available at NAR Online.

SUPPLEMENTARY DATA
