# Supplementary material for: Improving small RNA-seq by using a synthetic spike-in set for size-range quality control together with a set for data normalization
Source: Nucleic Acids Res. 2015 Apr 13;43(14):e89. doi: 10.1093/nar/gkv303 (PMC4538800; doi:10.1093/nar/gkv303)
Supplement: SUPPLEMENTARY DATA [file supp_43_14_e89__index.html]

Improving small RNA-seq by using a synthetic spike-in set for size-range quality control together with a set for data normalization — SUPPLEMENTARY DATA 

# Improving small RNA-seq by using a synthetic spike-in set for size-range quality control together with a set for data normalization

## SUPPLEMENTARY DATA

**Files in this Data Supplement:**

- SUPPLEMENTARY DATA
- SUPPLEMENTARY DATA
- SUPPLEMENTARY DATA
- SUPPLEMENTARY DATA
