# Supplementary material for: Improving small RNA-seq by using a synthetic spike-in set for size-range quality control together with a set for data normalization
Source: Nucleic Acids Res. 2015 Apr 13;43(14):e89. doi: 10.1093/nar/gkv303 (PMC4538800; doi:10.1093/nar/gkv303)
Supplement: SUPPLEMENTARY DATA [file supp_gkv303_nar-00684-met-k-2015-File007.docx]

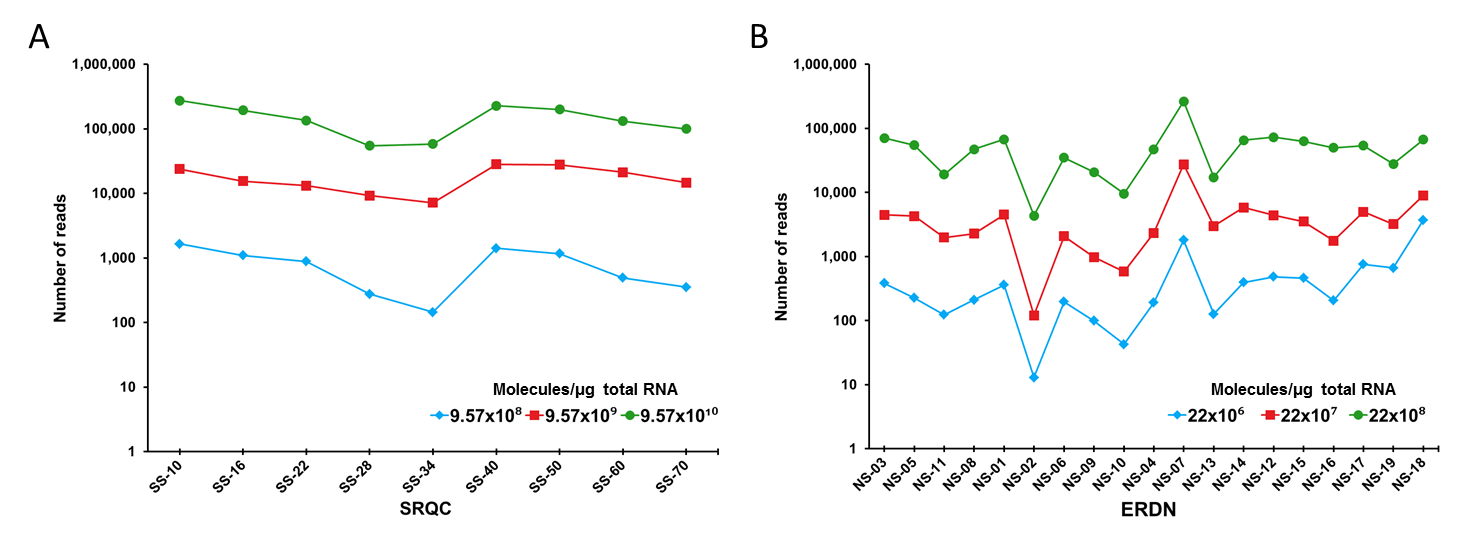


**Supplementary Figure 1.1.** *Sequencing efficiency of the individual spike-in oligonucleotides.* NGS results of three increasing concentrations of the size (A) and normalization (B) spike-in control sets. All spike-in controls were used at an equal concentration (blue, red, and green) in each test experiment that contained an identical miRNA background. Spike-in controls SS-19 and SS-25 were not ready at the time of these experiments. The NGS-library preparation was done without any size selection.

**Supplementary Table 1.2.** *Concentration-correction table for the size spike-in controls*Name: short name for each size spike-in control; molec/µl stock: the number of molecules per µl at which each oligonucleotide was supplied (100 µmol/l); DF: the dilution factor that was needed to obtain an adequate final number of reads; CF: correction factor. An additional dilution factor that is needed to obtain a similar number of reads for all size spike-ins. Spike-in SS-19 is used as a reference (CF=1) to which the other spike-ins are scaled; Final DF: the final dilution factor, equaling DF × CF; molec/µl final: the number of molecules in the final spike-in mix, of which 1 μl is to be added per 5 μg of total RNA.

**Supplementary Table 1.3.** *Concentration-correction table for the normalization spike-in controls*

Name: short name for each normalization spike-in control; molec/µl stock: the number of molecules per µl at which each oligonucleotide was supplied (100 µmol/l); DF: the dilution factor that is needed to obtain 2-fold decreasing read counts from top to bottom of the list; CF: correction factor. An additional dilution factor that is needed to correct for differences in sequencing efficiency. The average of all ERDN read counts is used as a reference (CF=1) to which the other spike-ins are scaled; Final DF: the final dilution factor, equaling DF × CF; molec/µl final: the number of molecules in the final spike-in mix, of which 1 μl is to be added per 5 μg of total RNA.
