# Supplementary material for: Improving small RNA-seq by using a synthetic spike-in set for size-range quality control together with a set for data normalization
Source: Nucleic Acids Res. 2015 Apr 13;43(14):e89. doi: 10.1093/nar/gkv303 (PMC4538800; doi:10.1093/nar/gkv303)
Supplement: SUPPLEMENTARY DATA [file supp_gkv303_nar-00684-met-k-2015-File008.docx]

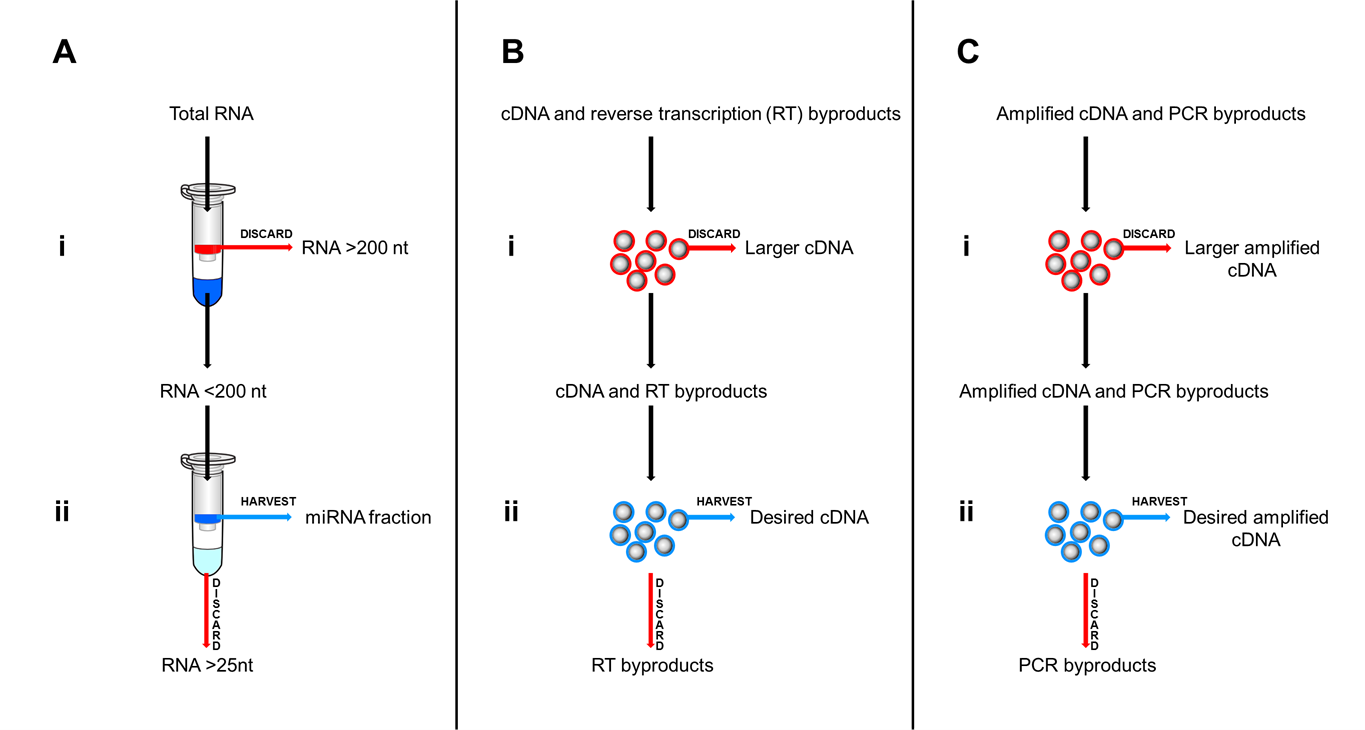


**Supplementary Figure 2.1**. *Outline of the principle of size selection during Ion Torrent sRNA-seq library preparation.* A graphical representation is shown of the differential binding of small (blue) and large RNA/cDNA (red) to spin columns or nucleic acid binding beads during (A) small RNA enrichment, (B) cDNA size selection and (C) amplified cDNA size selection. In the first step (i), large RNA or cDNA is captured on spin columns or beads using low ethanol concentration and subsequently discarded. In the second step (ii), the flow through containing smaller RNA or cDNA is bound to a new column or beads by increasing the ethanol concentration. For small RNA enrichment the ethanol concentration in the second step is chosen such that most RNA >15 nt will be bound to the second column and eluted. During cDNA and amplified cDNA size selection, the second ethanol concentration is only slightly increased to prevent binding of adapters/primers and exclusively enrich for library products with a miRNA-sized insert.


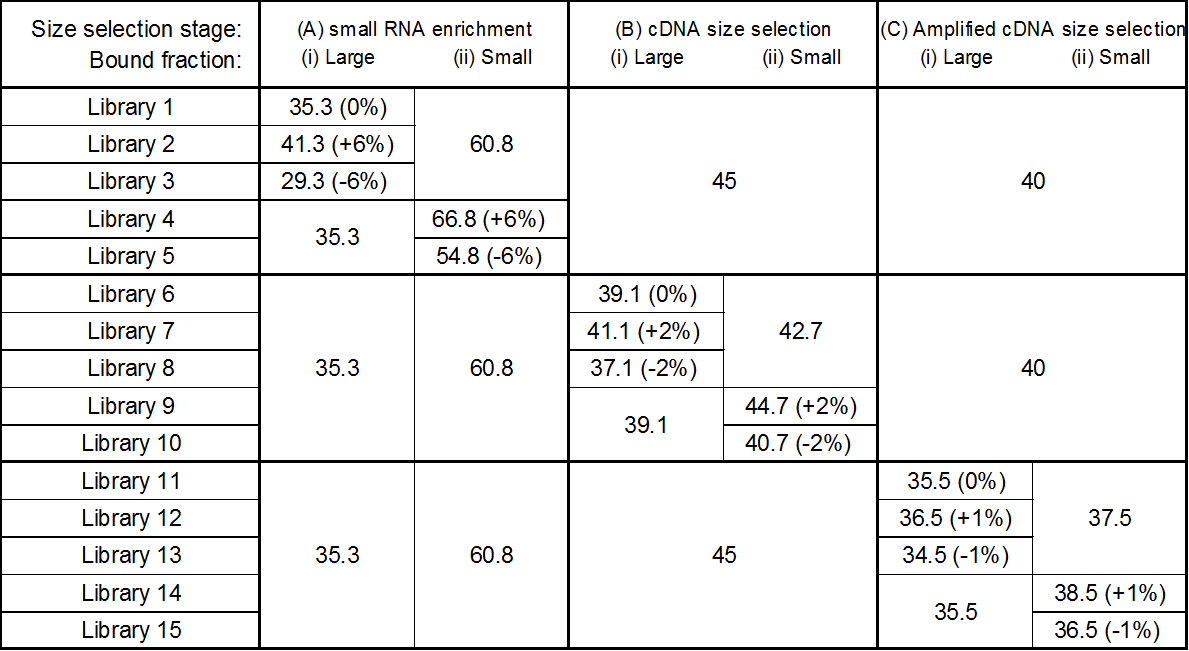


**Supplementary Table 2.2.** *Experiment scheme to investigate the effect of ethanol concentration on the sRNA-seq size range.* The effect of an increase or decrease in ethanol concentration of 1, 2 and 6% compared to the standard concentration (0%) was respectively tested for the three size-selective stages (A-C, see scheme above) in sRNA-seq. For each stage under study, there was no size selection at the other stages. Within each size selective stage, large RNA/cDNA depletion (i) and subsequent small RNA/cDNA binding (ii) were independently varied. If step (i) was altered then step (ii) was performed using the standard concentration and vice versa. Values are final ethanol concentrations in % (v/v).


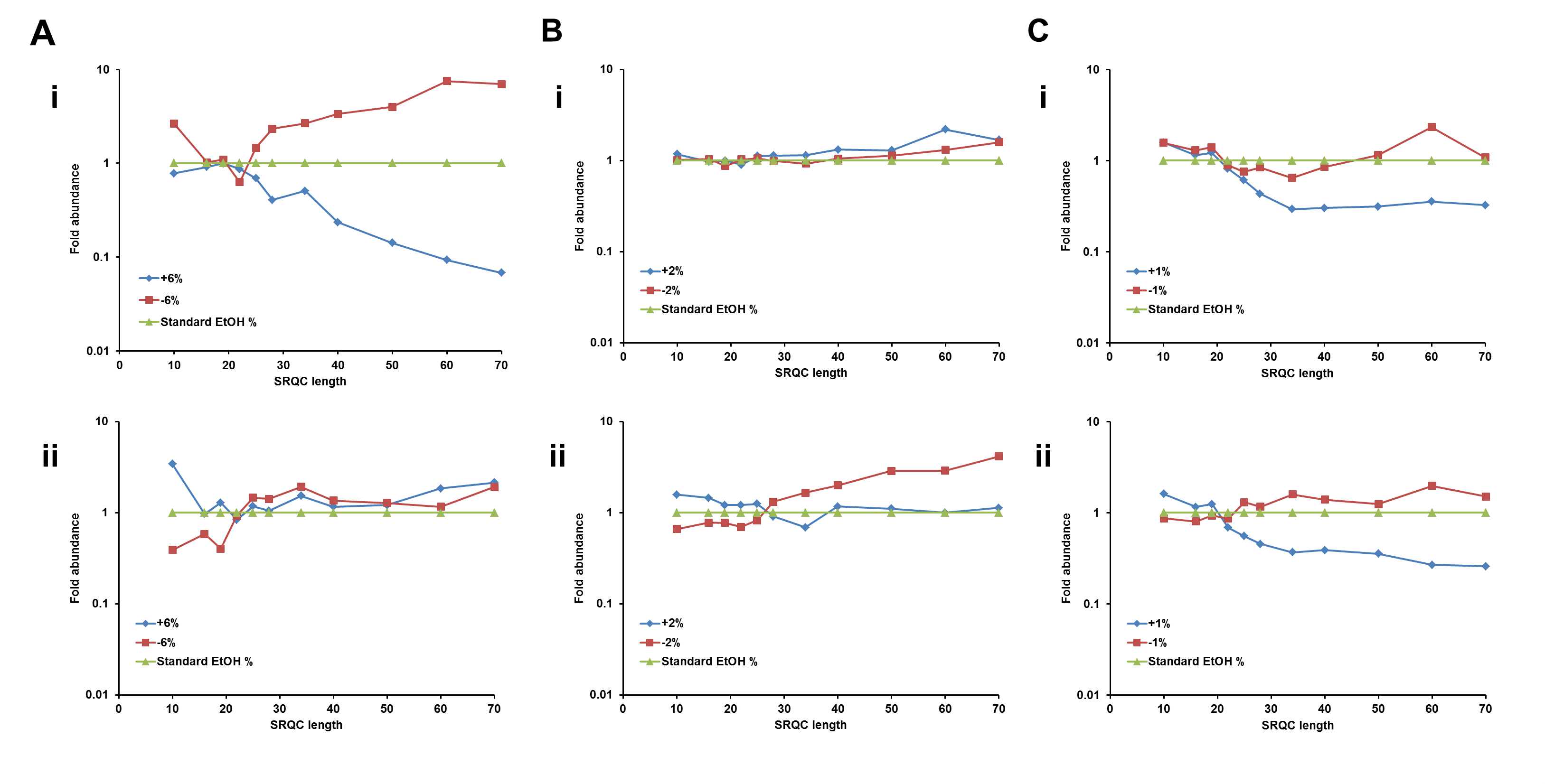


**Supplementary Figure 2.3.** *Ethanol-concentration dependent size selection in sRNA-seq.* The effect of increased (blue) and decreased (red) ethanol concentrations on the size range of the Ion Torrent sRNA-seq procedure is shown for small RNA enrichment (A), cDNA size selection (B), and amplified cDNA size selection (D). The number of reads that mapped to each size spike-in was normalized to the number of total mapped miRNA reads per sample. The size selectivity response to variations in ethanol concentration is plotted as the ratio of the number of reads for each size spike-in (SS-10 to SS-70) over the number of reads observed for that spike-in using standard protocol ethanol concentrations (Table S2.2). A ratio of one thus indicates that size range was not affected. The large RNA/cDNA removal (i) and subsequent small RNA/cDNA binding (ii) steps were tested separately.
