# Supplementary material for: Improving small RNA-seq by using a synthetic spike-in set for size-range quality control together with a set for data normalization
Source: Nucleic Acids Res. 2015 Apr 13;43(14):e89. doi: 10.1093/nar/gkv303 (PMC4538800; doi:10.1093/nar/gkv303)
Supplement: SUPPLEMENTARY DATA [file supp_gkv303_nar-00684-met-k-2015-File009.docx]

**
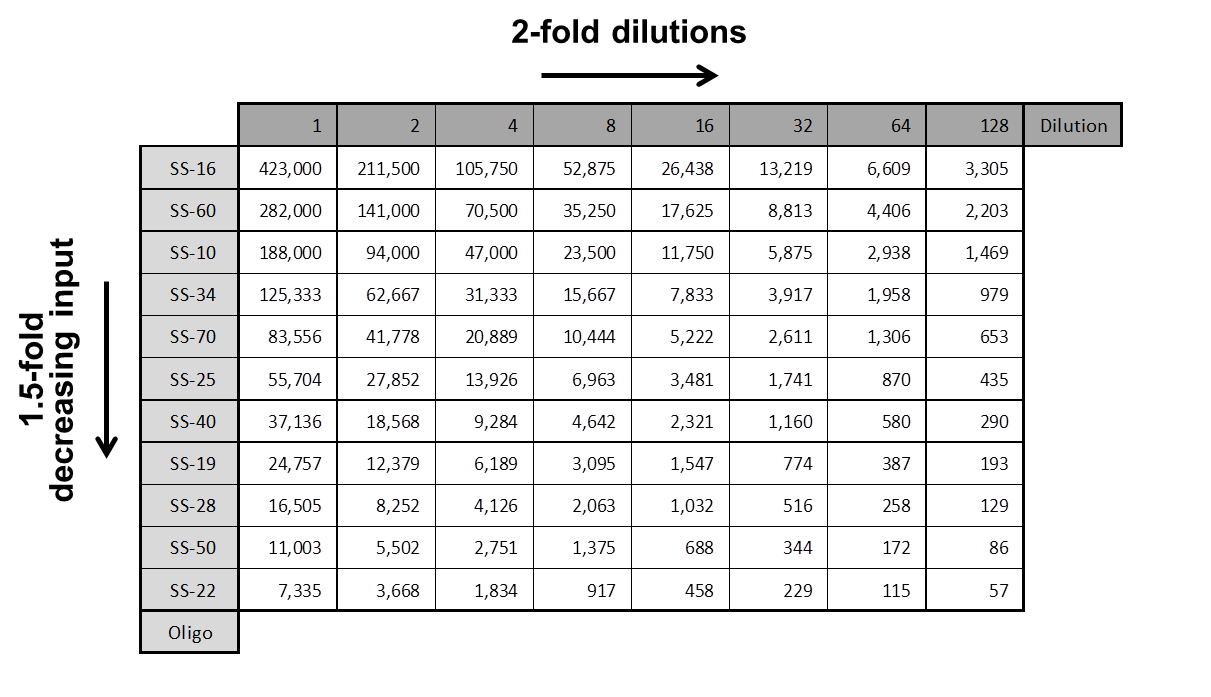
**

**Supplementary Table 3.1.** *Experiment scheme to investigate the performance of data normalization with ERDN controls.* Predetermined fold-change oligos (SS-10 to SS-70, serving here as spike-ins with known differences in RNA input between samples) were randomized and diluted according to the table above using 1.5-fold differences in the number of input molecules between consecutive oligos. From this mix, 2-fold to 128-fold dilutions were made by serial dilution. Values are in zeptomole/μg total RNA. Each fold-change mix was then combined with a fixed amount of the ERDN mix and added to equal aliquots taken from a single batch of adult male zebrafish total RNA.
